# Effects of Dietary Cinnamaldehyde Supplementation on Growth Performance, Serum Antioxidant Capacity, Intestinal Digestive Enzyme Activities, Morphology, and Caecal Microbiota in Meat Rabbits

**DOI:** 10.3390/ani15152262

**Published:** 2025-08-01

**Authors:** Dongjin Chen, Yuxiang Lan, Yuqin He, Chengfang Gao, Bin Jiang, Xiping Xie

**Affiliations:** 1 Institute of Animal Husbandry and Veterinary Medicine, Fujian Academy of Agricultural Sciences, Fuzhou 350013, China; chendongjin@faas.cn (D.C.);; 2 College of Animal Science, Fujian Agriculture and Forestry University, Fuzhou 350003, China; 3 Fujian Key Laboratory of Animal Genetics and Breeding, Fuzhou 350013, China; 4 Key Laboratory of Fujian Universities Preventive Veterinary Medicine and Biotechnology, Longyan University, Longyan 364012, China

**Keywords:** meat rabbit, cinnamaldehyde, growth performance, digestive enzymes, intestinal morphology, caecal microbiota

## Abstract

Cinnamaldehyde (CA) is a potential substitute for antibiotic growth promoters in animal breeding. In this study, we investigated its effects as a dietary supplement on growth performance, serum antioxidant capacity, intestinal digestive enzyme activities, intestinal morphology, and caecal microbiota in meat rabbits. Weaned meat rabbits (*n* = 450) were randomly assigned to five groups, Groups A, B, C, D, and E, and fed 0, 50, 100, 150, and 200 mg/kg CA diets, respectively, for 47 days. Biological samples including serum (antioxidants), duodenal/caecal content (enzymes), intestinal tissue (morphology), and caecal digesta (microbiota) were collected at day 47 postweaning for analysis. Groups C and D showed significantly higher final body weights than Group A, with Group D (150 mg/kg CA) demonstrating superior growth performance including 11.73% longer duodenal villi (*p* < 0.05), 28.6% higher microbial diversity (*p* < 0.01), and 62% lower diarrhoea rate versus controls. Digestive enzyme activity as well as serum antioxidant capacity increased with increasing CA dose, Microbiota analysis revealed CA increased fibre-fermenting Oscillospiraceae (+38%, *p* < 0.01) while reducing Ruminococcaceae (−27%, *p* < 0.05). Thus, dietary CA supplementation at 150 mg/kg was identified as the optimal CA dose for improving meat rabbit production. These findings highlight CA as a functional feed additive for promoting sustainable rabbit production.

## Simple Summary

The restriction of antibiotic growth promoters (AGPs) in animal farming has driven the search for sustainable alternatives. Cinnamaldehyde (CA), a phytogenic compound, shows promise due to its antimicrobial, antioxidant, and gut health benefits. This study aimed to investigate the effects of dietary CA supplementation on the growth performance, serum antioxidant capacity, intestinal digestive enzyme activities, morphology, and caecal microbiota of meat rabbits. Our findings revealed that dietary CA supplementation not only exerted positive effects on the growth performance of the test animals but significantly improved intestinal digestive enzyme activities, intestinal morphology, and caecal microbiota regulation, reducing diarrhoea and mortality rates. Further, the diet containing 150 mg/kg CA showed optimal effects. Therefore, the use of CA as an efficient and sustainable antibiotic to promote meat rabbit production is highly recommended.

## 1. Introduction

The worldwide restriction of the addition of antibiotics in animal breeding since 2020 has compelled the livestock sector to fundamentally reorient production strategies [1]. This regulatory transition to antibiotic-free animal production has also driven innovation in sustainable husbandry methodologies [2]. However, it poses a critical challenge with respect to maintaining industrial-scale productivity and meeting more stringent food safety requirements [3]. To address this issue, we propose an integrated framework comprising three synergistic domains. The first involves the systematic optimisation of husbandry ecosystems via precision environmental control coupled with enhanced biosecurity measures (i.e., all-in/all-out systems and advanced disinfection protocols) [4]. The second domain comprises proactive health-management systems that integrate precise vaccination programs based on pathogen prevalence mapping [5], and the third involves innovative nutritional interventions based on nutrigenomics-guided feed formulations that emphasise the strategic incorporation of functional additives and synergistic use of microbiota modulators [6,7,8,9,10].

China dominates global rabbit production and trade, accounting for 27% of worldwide meat rabbit exports [11]. Further, the rabbit production industry in China has emerged as a strategic component of China’s rural revitalisation framework, contributing significantly to poverty alleviation efforts, while serving as a crucial driver for restructuring the livestock industry and sustainable agricultural development [12]. However, this sector faces the critical challenge of weaning-associated diarrhoea in young rabbits. As monogastric herbivores with immature digestive systems, rabbits experience severe weaning stress that frequently induces gastrointestinal lesions, diarrhoea, and subsequent mortality [13], and this pathology accounts for substantial economic losses, with epidemiological data revealing that more than 70% of deaths within the baseline mortality rate (20%) in rabbit flocks stems from diarrhoeal diseases. Additionally, severely affected farms report mortality rates of 50–70%, with diarrhoea accounting for >80% of the recorded cases [14,15]. Weaning in rabbits triggers gastrointestinal (GI) disorders through four key factors: immature gut development, dietary changes, stress responses, and microbial imbalance [16,17]. While thymol and eugenol have shown moderate benefits [18,19], cinnamaldehyde (CA) offers superior stability and efficacy [20]. The need for alternatives is urgent, with 68% of Chinese rabbit farms reporting antibiotic-resistant *E. coli* [21].

The intensified search for novel antibiotic alternatives in animal nutrition research has led to the identification of plant essential oils as promising candidates. These phytogenic compounds, extracted from various plant organs, contain multiple bioactive constituents that enhance immune function, disease resistance, and production efficiency, while improving livestock product quality [22]. Among these, cinnamaldehyde (CA), a phenolic aldehyde obtained via CO_2_ supercritical fluid extraction from the bark of *Cinnamomum* spp. or via synthetic pathways, has potential as an antibiotic alternative [23]. With molecular formula C_9_H_8_O (MW: 132.16), CA exhibits broad-spectrum antimicrobial, antioxidant, and immunomodulatory properties [24,25] via mechanisms similar to those of conventional antibiotics [26]. The US FDA categorises CA as “generally recognised as safe (GRAS)” for food applications [27], and it has also been listed in China’s National Food Safety Standard. Previous studies have also shown that CA demonstrates efficacy in multiple livestock species, including swine [28], poultry [29,30], ruminants [31], and aquaculture species [32].

Therefore, given the dual functionality of CA as both an immune enhancer and an anti-inflammatory agent, this paper systematically investigates the effects of CA on the incidence of diarrhoea and growth performance of meat rabbits. We also elucidated the mechanisms underlying its modulation of intestinal health and identified the optimal dietary concentration. The findings of this study may provide evidence-based recommendations for the use of CA as an alternative to antibiotics for promoting growth in meat rabbit farming.

## 2. Materials and Methods

### 2.1. Experimental Materials

CA (20% active compound in dextrin carrier) was provided by Jiangsu Kangya Biotechnology Co., Ltd. (Jingjiang, China). A total of 450 Ira meat rabbits (*Oryctolagus cuniculus*; age, 35 days; initial body weight, 1095.42 ± 30.57 g) were sourced from Nanping Puyang Rabbit Industry Co., Ltd. (Nanping, China).

### 2.2. Experimental Design and Feeding Management

All experimental procedures were performed under controlled environmental conditions at the Intelligent Rabbit Farming Facility of Fujian Academy of Agricultural Sciences. Using a completely randomised design, the rabbits were assigned to five diet groups (A–E; *n* = 90 per group). The rabbits Groups A to E were fed the basal diet supplemented with 0, 50, 100, 150, and 200 mg/kg of CA, respectively. No additional medications or additives were included in either the control or treatment diets besides the specified cinnamaldehyde supplementation.

The 90 rabbits in each group (45 males and 45 females) were further distributed across three replicates (*n* = 30 rabbits/replicate, with 2 rabbits/cage). Standardised cage dimensions (L × W × H: 42 × 50 × 35 cm) were maintained throughout the 47-day experimental period, which comprised a 5-day initial acclimation followed by a 42-day investigation phase. Ad libitum feeding protocols, comprising pelletised diet and water, were implemented for the rabbits in all the treatment groups under standardised housing parameters. The facility maintained a temperature of 20–24 °C and a relative humidity of 60–65%. Ventilation was provided through a mechanical system ensuring air exchange rates of 10–15 air changes per hour. Further, the feeding regimes and immunisation protocols were in accordance with standard commercial practices. The immunisation schedule included the following: at 35 days of age, vaccination against rabbit haemorrhagic disease (RHDV) using an inactivated vaccine (1 mL subcutaneously); at 42 days of age, vaccination against *Pasteurella multocida* (1 mL intramuscularly). Booster doses were administered at 14-day intervals for both vaccines. Health monitoring was conducted daily, and no antibiotics or additional medications were administered during the experimental period to avoid confounding effects. The feed ingredients and nutritional components detailed in Table 1 were formulated primarily according to the nutritional requirements for fattening rabbits established by the National Research Council (NRC) of the United States (1977), with adjustments informed by De et al. [33] to address contemporary breeding conditions.

To ensure dietary consistency across all experimental groups, feed preparation was conducted in three standardised phases every 15 days. Pulverisation Phase: The same basal diet formulation (composition detailed in Table 1) was uniformly ground to a fine powder (particle size < 500 μm) using a centrifugal mill. Cinnamaldehyde Incorporation and Pelletisation: In the treatment groups, the powdered basal diet was blended with cinnamaldehyde at graded concentrations for 20 min in a double-cone mixer (RPM = 35). The control group received basal powder without additive. All mixtures were moistened with water and pelleted (Φ3 × 10 mm pellets) via a ring-die pelletiser (60 °C). Final Feed Formulation: Final feed was dried (40 °C, 12 h) to moisture content <11% and stored in a cool and dry place.

### 2.3. Growth Performance Measurement

Individual body weights were recorded at 08:00 after overnight fasting (20:00–08:00), with pre-experimental (initial body weight, IBW) and postexperimental (final body weight, FBW) measurements constituting core growth evaluation parameters. Throughout the experimental period, the rabbits were monitored daily for diarrhoea and mortality. Additionally, feed consumption parameters, including daily provision quantities, residual amounts, and spillage (feed spillage was quantified gravimetrically using preweighed trays), was recorded, and used to calculate key growth performance parameters as follows:

Actual Intake = (Feed Provided) − (Residual Feed in Feeder) − (Weighed Spillage)(1)

Average daily gain (ADG, g/d) = (FBW − IBW)/experimental duration (days)(2)

Average daily feed intake (ADFI, g/d) = total feed consumption/(experimental duration × number of rabbits)(3)

Feed conversion ratio (FCR) = ADFI/ADG(4)

Diarrhoea rate (%) = (number of diarrhoeic rabbits/total number of rabbits) × 100(5)

Mortality rate (%) = (number of deceased rabbits/total number of rabbits) × 100(6)

The experimental protocol was designed to ensure accurate assessment of growth parameters while maintaining animal welfare standards.

Through comparative analysis of production performance, the control group (A) and the treatment groups with the best and second-best performance (D and C, respectively) were selected for subsequent experimental testing.

### 2.4. Detection of Serum Antioxidant Capacity

The sterile collection of venous blood (2 mL) from the ear veins of the experimental rabbits was performed after the experiment (*n* = 18 per group). These aliquots were then immediately placed in sterile vacutainer tubes for ambient temperature coagulation over a period of 30 min. Subsequently, the samples were subjected to refrigerated centrifugation at 4 °C and 3000× *g* for 10 min, and the resulting serum fractions were carefully aliquoted into cryovials and preserved at −80 °C in ultralow-temperature conditions until biochemical analysis. Serum antioxidant capacity was assessed via the quantification of key enzymatic markers: superoxide dismutase (SOD), glutathione peroxidase (GSH-PX), catalase (CAT), and thioredoxin-dependent peroxidase (TPX) using a standardised ELISA methodology employing commercially available kits (Nanjing Jiancheng Bioengineering Institute, Nanjing, China) in strict accordance with established protocols.

### 2.5. Determination of Intestinal Digestive Enzyme Activity

On the final day of the experiment, rabbits (*n* = 6 per group) were euthanised humanely, and the caecum and duodenum were immediately dissected. The luminal contents were aseptically collected using sterile spatulas, transferred into 5 mL sterile cryovial tube, and snap-frozen in liquid nitrogen. Samples were stored at −20 °C until analysis to preserve enzymatic activity and microbial integrity. The activities of intestinal amylase (AMY), trypsin (TPS), and lipase (LPS) were determined via colorimetry using reagent kits produced by the Nanjing Jiancheng Bioengineering Institute with triplicate measurements. AMY activity was determined by the starch–iodine method (Kit C016-1-1) at 660 nm absorbance (U/L). TPS: p-Nitroaniline release from chromogenic substrate (Kit A080-2-1) was monitored at 405 nm (U/mL). LPS: Olive oil emulsion hydrolysis (Kit A054-1-1) was measured via fatty acid titration (U/L). Absorbance readings were performed using a BioTek microplate reader. Methodologies followed Yang et al. [34] with protocol adaptations.

### 2.6. Histomorphological Analysis of Intestinal Tissues

Following overnight fasting, 1 cm intestinal segments were collected from the mid-duodenal region (between the pylorus and duodenojejunal flexure) and mid-colonic portion of euthanised animals (*n* = 6 per group). Intestinal lumen contents were removed via gravity perfusion using sterile 0.9% NaCl solution (4 °C). Subsequently, the tissue samples were immediately placed in 10% neutral buffered formalin, fixed at 4 °C for 24 h, and then embedded in paraffin and stained with haematoxylin and eosin (H&E). Finally, using the object quantification module in the Image-Pro Plus 6.0 software, quintuplicate intact villus structures per sample were systematically identified based on length-to-width ratios >3:1 and apical membrane integrity. Villus height (from crypt neck to apical tip) and crypt depth (from base to crypt–villus junction) were measured in triplicate by blinded investigators, with the final values representing the arithmetic mean of three independent measurements.

### 2.7. Caecal Caecal Microbiota Analysis

A subset of rabbits from Groups A (control), C (100 mg/kg CA), and D (150 mg/kg CA) were selected for microbiota analysis (*n* = 6 per group). Groups B and E were excluded; as their growth performance and health metrics were intermediate between Groups A and C/D, excluding them allowed focused comparison of the control against optimal CA doses. Following a 12 h fasting period with ad libitum access to water, humane euthanasia was administered, and within 5 min postmortem, caecal contents were aseptically collected using sterile surgical instruments, immediately transferred into 5 mL cryovials (Corning Inc., Corning, NY, USA), rapidly frozen in liquid nitrogen, and stored in a validated ultralow-temperature freezer at −80 °C. Microbial genomic DNA was then isolated from 200 mg caecal-content aliquots using the DNeasy PowerSoil Pro Kit (Qiagen, Hilden, Germany), incorporating bead-beating homogenisation (FastPrep-24, MP Biomedicals, Santa Ana, CA, USA). To assess genomic DNA quality, two analytical approaches were employed: (1) electrophoretic resolution using 1% agarose matrix (5 V/cm, 45 min) to confirm structural integrity, and (2) spectrophotometric quantification (NanoDrop OneC, Thermo Scientific, Waltham, MA, USA) with stringent purity thresholds (260/280 nm absorbance ratio: 1.8–2.0; 260/230 nm >2.0) to determine DNA concentration. The hypervariable V3–V4 regions of bacterial 16S rRNA genes were amplified using fusion primers, *338F (5′-ACTCCTACGGGAGGCAGCAG-3′)* and *806R (5′-GGACTACHVGGGTWTCTAAT-3′)*, with Illumina adapter sequences. Further, an Illumina MiSeq PE300 platform from Shanghai Meiji Biomedical Technology Co., Ltd. (Shanghai, China) was used for sequencing. Raw reads were quality-filtered (fastp v0.19.6; Q20 cutoff, 50 bp minimum length), merged (FLASH v1.2.7; 10 bp overlap, 0.2 mismatch ratio), and clustered into OTUs (UPARSE v7.1, 97% similarity), with chloroplast/mitochondrial sequences removed. Taxonomy was assigned (RDP Classifier v2.2; SILVA v138, 70% confidence), and sequences were rarefied to 20,000/sample (Good’s coverage > 99%).

Alpha diversity metrics (Shannon, Simpson, and Chao1 indices) were analysed using Kruskal–Wallis tests with Dunn’s post hoc correction for group comparisons. Beta-diversity was analysed using principal coordinate analysis (PCoA) based on weighted UniFrac distances with statistical significance assessed by ANOISM. Functional prediction (KEGG) and statistical testing (PERMANOVA, Kruskal–Wallis, FDR correction) were performed using QIIME2 (v2020.11) and R (v4.0.3; phyloseq, ggplot2) [35].

### 2.8. Data Statistical Analyses

Raw data were processed using Excel 2020 and analysed using SPSS software version 20.0. Comparisons of group means were realised via one-way ANOVA and Tukey’s test. *p* < 0.05 indicated significant differences and *p* < 0.01 indicated extremely significant differences.

## 3. Results

### 3.1. Growth Performance

The effects of dietary CA supplementation on the growth performance parameters of the meat rabbits are summarised in Table 2. Comparative analysis revealed that CA administration had no significant effect (*p* > 0.05) on ADFI or feed conversion ratio (F/G) relative to the control group. However, significant improvements in growth metrics were observed in the CA-supplemented treatment groups at the end of the treatment period. Specifically, Groups C and D demonstrated statistically significant increases in FBW compared with the control group (*p* < 0.05). Notably, Group D exhibited enhanced growth performance, with a significantly higher ADG than that observed for Group A (*p* < 0.05). Regarding health parameters, Group D showed superior outcomes, with significantly reduced diarrhoea incidence (DR) and mortality rates (MR) compared with all the other experimental groups (*p* < 0.05). Further, Group C displayed intermediate CA efficacy, showing a significantly lower mortality rate than both the Group A and Group E (*p* < 0.05). These findings suggested a dose-dependent relationship between CA supplementation and health improvement indicators in the experimental animals. However, excessive addition resulted in negative side effects, as observed in Group E.

### 3.2. Serum Antioxidant Capacity

The effects of CA on the serum antioxidant capacity and lipid indices of rabbits are presented in Table 3. The concentrations of serum SOD, GSH-PX, CAT, and TPX increased significantly with increasing dietary CA supplementation (*p* < 0.05). All CA-supplemented groups showed significant improvements (*p* < 0.05) in antioxidant capacity compared with the control (Group A), with clear dose-dependent trends. SOD activity increased progressively from 56.09 U/mL (Group A) to 74.20 U/mL (Group D), representing a 32.4% enhancement at the optimal dose (150 mg/kg CA). GSH-PX levels demonstrated a similar pattern, with Group D (150 mg/kg) showing the peak activity (258.70 U/mL), 19.4% higher than control. Group C (100 mg/kg) exhibited intermediate values (239.28 U/mL). CAT and TPX activities followed identical trends, with maximal improvements observed at 150 mg/kg CA (CAT: +19.5%; TPX: +28.6% vs. control).

### 3.3. Intestinal Digestive Enzyme Activity

As shown in Table 4, comparative analysis of intestinal enzymes revealed significant dose-dependent enhancements, with duodenal trypsin activity increasing by 30.1% (286.26 U/mL) in Group C and 58.9% (349.79 U/mL) in Group D compared with controls (220.10 U/mL, *p* < 0.001), while caecal amylase showed a progressive 44.8% increase from Group A (65.39 U/L) to Group D (94.66 U/L). Lipase activity demonstrated maximal improvement at the 150 mg/kg CA dose, with Group D exhibiting significantly elevated activity in both intestinal segments (duodenum: +52.4%; caecum: +43.3%) relative to control values (*p* < 0.001).

### 3.4. Intestinal Morphology Analysis

**Figure 1:**
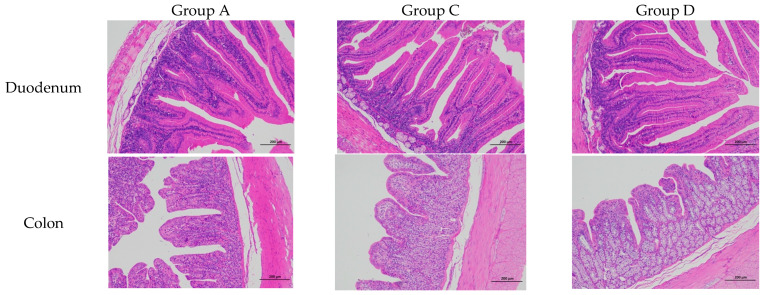
Effects of CA on intestinal tissue on morphology in rabbits (100×). Representative haematoxylin-and-eosin-stained sections of duodenum (upper panel) and colon (lower panel) from Groups A (control), C (100 mg/kg CA), and D (150 mg/kg CA). Scale bars = 200 μm.

CA supplementation significantly modulated duodenal mucosal architecture in the test animals (Table 5; Figure 1). Morphometric analysis demonstrated significant improvements in intestinal architecture, with Group D (150 mg/kg CA) showing an 11.7% increase in duodenal villus height (1111.80 μm vs control 994.99 μm, *p* < 0.05) compared with the nonsignificant improvement in Group C (1003.48 μm), while also achieving the highest V/C ratio (9.58 vs. 8.84 in controls), indicating enhanced intestinal absorptive capacity at the optimal supplementation level.

### 3.5. Caecal Microflora Profiling

#### 3.5.1. Alpha Diversity of Caecal Flora in Rabbits Supplemented with CA

V3–V4 hypervariable high-throughput sequencing revealed a dose-dependent modulation of caecal microbial diversity in the test animals owing to CA supplementation (Table 6). From a total of 18 caecal samples (*n* = 6 per group), we obtained 842,981 quality-filtered sequences (mean length = 409.38 bp) showing valid base calling accuracy >99.97%. Group D exhibited 19.4% higher Good’s coverage (99.51% in Group D vs. 99.28% in Group A, *p* < 0.05) and 28.6% greater observed species (Sobs = 941.83 vs. 871.17 in Group A, *p* < 0.05). The Chao richness estimator confirmed enhanced species diversity in Group D relative to Group A (1063.20 vs. 1018.50, *p* < 0.05). Notably, group C showed an elevated Simpson index (0.03 ± 0.00) relative to Groups A and D (0.02 ± 0.00, *p* < 0.05).

**Figure 2:**
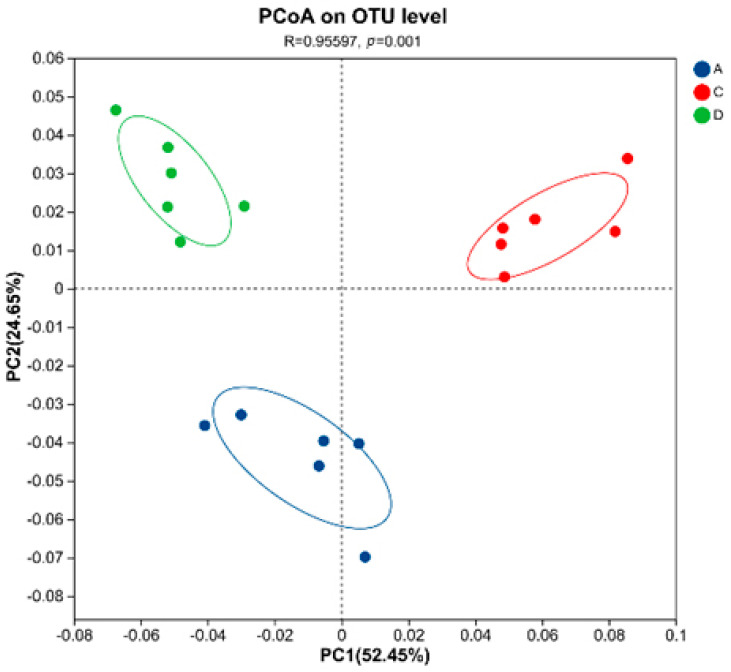
Principal coordinate analysis (PCoA) of caecal microbial communities.

Beta-diversity analysis based on NR species annotation revealed significant treatment-induced shifts in microbial community structure (R = 0.956, *p* = 0.001), with PCoA demonstrating clear group segregation along the primary axes, PC1 (52.45% variance) and PC2 (24.65% variance), collectively explaining 77.10% of total variation. Group A samples clustered distinctly in negative PC1 space (−0.04 to −0.08), while CA-supplemented groups (C/D) occupied positive PC1 coordinates (0.04 to 0.10), showing minimal cluster overlap. PERMANOVA confirmed strong group differentiation (*p* = 0.001) with significant dispersion effects (*p* < 0.05), particularly highlighting the most pronounced compositional differences in the 150 mg/kg CA group (D) compared with control (Figure 2).

The X and Y axes represent two selected principal axes, and the percentage represents the explanatory value of the principal axes for the differences in sample composition; the scales on the X and Y axes are relative distances and have no practical significance; differently coloured or shaped points represent samples from different groups; and the closer two sample points are, the more similar the species composition of the two samples is.

#### 3.5.2. Effects of CA on the Composition and Relative Abundance of Rabbit Caecal Flora at Phylum, Family, and Genus Levels

**Figure 3:**
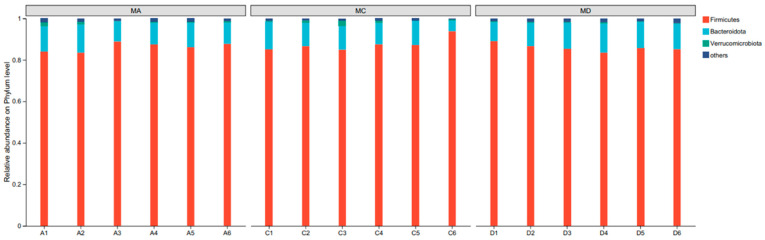
Phylum-level abundance of caecal microbiota in different diet groups. Relative abundance of major bacterial phyla (>1% total abundance) across treatment groups (*n* = 6 per group). Firmicutes and Bacteroidetes were the dominant phyla. No significant differences were observed among groups at this taxonomic level (*p* > 0.05).

The intestinal flora of the experimental rabbits predominantly comprised Firmicutes, Bacteroidoates, and Verrucomicrobiota, accounting for 86.57%, 11.23%, and 0.76% of the total bacteria population, respectively (Figure 3). Thus, there were no significant differences among the groups at the phylum level (*p* < 0.05).

**Figure 4:**
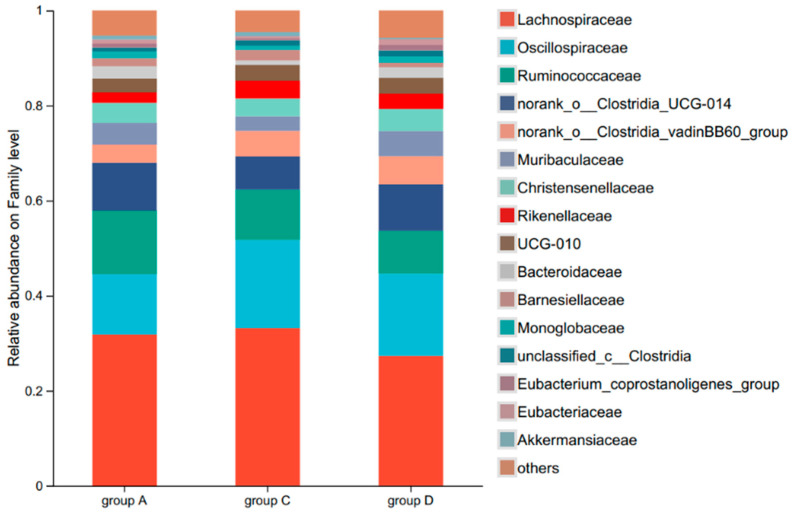
Family-level abundance of caecal microbiota in the different diet groups. Relative abundance (%) of bacterial families (>1% total abundance) in Groups A (control, 0 mg/kg CA), C (100 mg/kg CA), and D (150 mg/kg CA) (*n* = 6 per group).

At the family level, the caecal flora of the different groups were mainly composed of Trichophylidae, Polyphylidae, Rumenococcaceae, norank_o__Clostridia_UCG-014, norank_o__Clostridia_vadin, BB60_group, Muribaculaceae, Christensenellaceae, and Rikenellaceae, which accounted for 30.66%, 16.18%, 11.01%, 9.05%, 5.01%, 4.30%, 4.23%, and 3.11% of the total microbial abundance, respectively (Figure 4).

**Figure 5:**
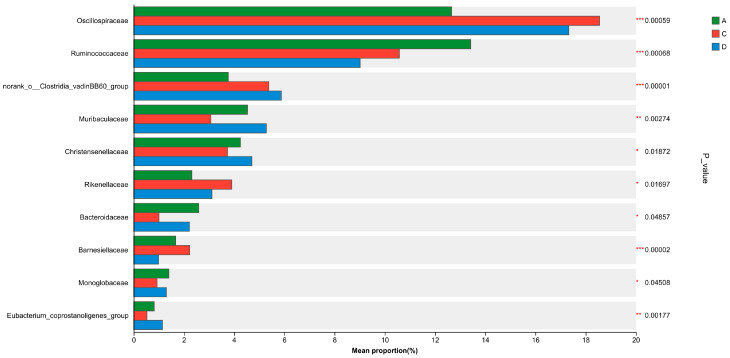
Differential abundance of caecal microbiota at the family level in response to dietary cinnamaldehyde (CA) supplementation. A bar chart displays the differences in the average relative abundance of the same species among Groups A (control, 0 mg/kg CA), C (100 mg/kg CA), and D (150 mg/kg CA) (*n* = 6 per group) and indicates whether the differences are significant (*p*-values, asterisks represent significant differences), intuitively demonstrating the significant differences in the abundance of a given species among multiple different groups. Figure note: the Y-axis represents the species names at a certain taxonomic level, the X-axis represents the average relative abundance of different groups of species, and differently coloured columns represent different groups. On the far right is the *p*-value, * 0.01 < *p* ≤ 0.05, ** 0.001 < *p* ≤ 0.01, *** *p* ≤ 0.001.

Comparative analysis of caecal microbiota revealed distinct taxonomic shifts among the experimental groups at family level. Groups C and D exhibited significant increases in the relative abundances of Oscillospiraceae and norank_o__Clostridia_vadinBB60_group relative to Group A (*p* < 0.01). Conversely, their relative abundances for Ruminococcaceae were significantly lower than that observed for Group A (*p* < 0.01). Further, Group D demonstrated unique microbial characteristics, showing a relative abundance for Muribaculaceae that exceeded those for both Groups A (*p* < 0.01) and C (*p* < 0.05). Similarly, Group D exhibited a significantly higher relative abundance of Christensenellaceae than Group C (*p* < 0.05), indicating CA’s dose-dependent modulation of gut microbiota composition (Figure 5).

**Figure 6:**
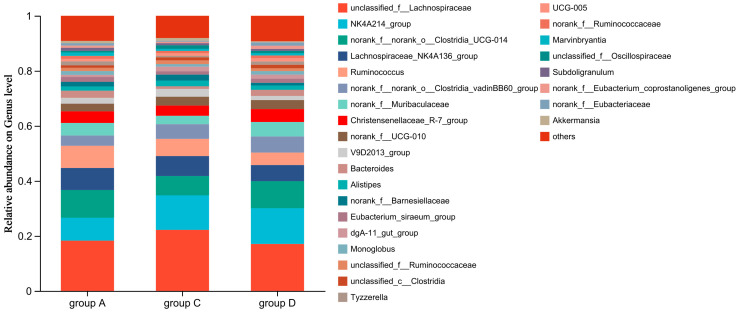
Genus-level relative abundance of caecal microbiota in different diet groups. The bar plot shows the relative abundance (%) of dominant bacterial genera (>1% total abundance) in Groups A (control, 0 mg/kg CA), C (100 mg/kg CA), and D (150 mg/kg CA) (*n* = 6 per group).

Genus-level analysis identified five predominant taxa constituting the core caecal microbiota across the experimental cohorts. The microbial composition was characterised by *unclassified_f__Lachnospiraceae* (19.09%), *NK4A214_group* (11.29%), *norank_f__norank_o__Clostridia_UCG-014* (9.05%), *Lachnospiraceae_NK4A136_group* (7.06%), and *Ruminococcus* (6.28%). Collectively, these taxa represented 52.77% of total microbial abundance, and their individual contributions were ranked as shown in Figure 6.

**Figure 7:**
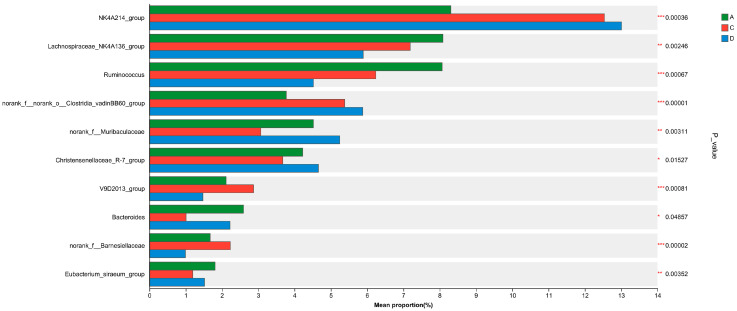
Genus-level differences in caecal microflora composition among the different diet groups. A bar chart displays the differences in the average relative abundance of the same species among different groups and indicates whether the differences are significant (*p*-value values, asterisks represent significant differences), intuitively demonstrating the significant differences in the abundance of a given species among multiple different groups. Figure note: the Y-axis represents the species names at a certain taxonomic level, the X-axis represents the average relative abundance of different groups of species, and differently coloured columns represent different groups. On the far right is the *p*-value, * 0.01 < *p* ≤ 0.05, ** 0.001 < *p* ≤ 0.01, *** *p* ≤ 0.001.

Caecal microbiota quantification demonstrated differential enrichment patterns across experimental cohorts (Figure 7). Groups C and D exhibited significantly elevated proportions of *NK4A214_group* (Lachnospiraceae family) and *norank_o_Clostridia_vadinBB60_group* compared with Group A (*p* < 0.01). Conversely, Group D showed marked depletion in two core commensals, *Lachnospiraceae_NK4A136_group* (27.14% reduction) and *Ruminococcus* (43.99% decrease), relative to group A (*p* < 0.01).

Alpha-diversity indices demonstrated significant improvements in Group D versus Group A (Shannon: 5.24 vs. 4.98, *p* = 0.018; Chao1: 1063 vs. 1018, *p* = 0.021; Kruskal–Wallis with Dunn’s correction). The analysis of caecal microbiota revealed significant dose-dependent alterations in microbial composition (*p* < 0.05, ANOISM). Beta-diversity analysis (weighted UniFrac) confirmed distinct clustering of microbial communities (R = 0.956, *p* = 0.001, ANOISM), with Group D samples showing the greatest separation from controls along PC1 (52.45% variance, *p* = 0.002). Groups C and D showed significantly increased *Oscillospiraceae and Clostridia_vadinBB60_group* (*p* < 0.01) but decreased Ruminococcaceae (*p* < 0.01) versus Group A, with Group D specifically exhibiting higher Muribaculaceae (vs. A *p* < 0.01, vs C *p* < 0.05) and Christensenellaceae (vs. C *p* < 0.05), demonstrating CA’s dose-dependent microbiota modulation at family level. Genus-level analysis revealed significant microbial shifts, with Groups C/D showing increased *NK4A214_group* (+28.6%, *p* < 0.01) and *Clostridia_vadinBB60_group* (+51.2%, *p* < 0.01) but decreased *Lachnospiraceae_NK4A136_group* (−27.1%, *p* < 0.01) and *Ruminococcus* (−44.0%, *p* < 0.01) versus Group A, demonstrating CA’s profound impact on core microbiota.

## 4. Discussion

### 4.1. Effects of Dietary CA Supplementation on the Growth Performance of Rabbits

CA, a principal bioactive C6–C3 phenylpropanoid that is phytochemically isolated from *Cinnamomum spp.*, shows broad-spectrum pharmacological efficacy owing to its antioxidant, anti-inflammatory, and antimicrobial properties and enhances gastrointestinal motility. As a phytogenic feed additive, its capacity to modulate nutrient utilisation efficiency and growth parameters has garnered considerable research interest in monogastric and ruminant nutrition [36]. The findings of the present study demonstrated species-specific growth-promoting effects consistent with the findings of previous studies. Song et al. [30] documented a 12% decline in FCR in heat-stressed ducks on a diet supplemented with 200 mg/kg microencapsulated CA. Similarly, Zhou et al. [37] observed a 40% decrease in the incidence of diarrhoea in weaned piglets administered 600 mg/kg of a CA-based antibiotic alternative. Studies in ruminants further corroborate these effects, with Zhang et al. [38] reporting a 15% improvement in milk yield in Holstein cows supplemented with 18 g/day CA, possibly owing to enhanced rumen volatile fatty acid profiles. Notably, our study revealed temporal specificity in the growth response of meat rabbits. Even though there were no significant differences among the treatment groups in terms of ADG and FCR during the 36–77-day growth phase (*p* > 0.05), final body weights in Groups C (2575.99 ± 12.76 g) and D (2624.42 ± 36.71 g) were significantly higher than that in Group A (2525.26 ± 9.62 g; *p* < 0.05). This delayed enhancement of growth suggested potential cumulative effects of CA on gut microbiome maturation and metabolic programming. The observed dichotomy in research outcomes may be attributed to three critical factors, namely dose–response relationships, delivery system optimisation, and stress modulation. For dose–response relationships, Yang et al. [39] reported null effects in calves administered suboptimal doses (<50 mg/kg BW), contrary to our effective regimen of 300–450 mg/kg. For delivery system optimisation, Chapman’s null findings in lactating cows [31] likely reflect inadequate rumen bypass, whereas our microencapsulated formulation ensured targeted intestinal delivery. For stress modulation, we observed that Group D exhibited a 62% decrease in DR and 75% lower MR than the control (*p* < 0.05). CA enhances gut barrier function by upregulating tight junction proteins (ZO-1, occludin) through TLR4/NF-κB inhibition [36] while selectively modulating gut microbiota to favour beneficial butyrate-producing bacteria (e.g., Oscillospiraceae) and suppressing pathogens [34]. These coordinated actions improve mucosal integrity [40] and explain the reduced diarrhoea incidence observed with CA supplementation in our study.

### 4.2. Effects of Dietary CA Supplementation on Serum Antioxidant Capacity of Meat Rabbits

Antioxidant capacity is an indicator of the amount and activity of antioxidants in the body. It also reflects the ability of the body to scavenge free radicals and reduce antioxidative stress and is an important indicator for assessing health status and disease risk [34]. El-Aziz et al. [24] showed that plant essential oils can improve antioxidant capacity. Similarly, the results of this study indicated that dietary CA supplementation enhanced antioxidant capacity in the test animals by increasing serum SOD, GSH-Px, CAT, and TPX levels. These findings are consistent with those reported by Luo et al. [28], who observed significantly increased GSH-Px levels (*p* < 0.05) in finishing pigs following dietary CA supplementation at 40 and 80 mg/kg. It has also been observed that lauric acid monoglyceride (350 and 500 mg/kg) and CA-complex plant essential oils can improve the antioxidant capacity of broiler [41]. Further, it has been shown that CA enhances antioxidant activity in rabbit erythrocytes [42] and regulates H_2_O_2_-induced skeletal muscle atrophy by ameliorating antioxidant defence systems [43]. The observed improvements in serum antioxidant markers (SOD: 74.20 U/mL in Group D vs. 56.09 U/mL in controls; CAT: 67.97 vs. 56.90 U/mL) exceeded the established reference ranges for healthy rabbits (SOD: 40–60 U/mL; CAT: 45-55 U/mL) [44], indicating CA’s potent antioxidant effects.

### 4.3. Effects of Dietary CA Supplementation on Intestinal Digestive Enzyme Activity

Gastrointestinal processing efficiency fundamentally depends on the activities of the catalytic triad, pancreatic amylase, protease, and lipase in hydrolysing complex nutrients into absorbable monomers. Yang et al. [34] demonstrated that dietary CA supplementation significantly improves intestinal digestive and absorptive functions in broiler chickens, as evidenced by enhanced brush border enzyme activity and nutrient transporter expression. This previous study highlighted the potential of CA as a phytogenic additive for optimising gut efficiency and nutrient utilisation in monogastric species. Cui et al. [41] also noted that dietary supplementation with 300 mg/kg CA significantly improves the activity of digestive enzymes (such as amylase and xylanase) in the rumen of fattening sheep. Notably, the dose-dependent effects of CA (optimal at 300 mg/kg) observed in this study underscore the importance of precise CA supplementation strategies. Our findings also indicated that dietary CA supplementation enhanced the activities of lipase, amylase, and trypsin in the small intestine and caecum of the test animals while promoting the digestion of nutrients, such as fat, dry matter, and protein, and ultimately improving growth performance. Digestive enzyme activities (amylase, trypsin, lipase) were normalised to total protein content (Bradford assay) and expressed as units per millilitre of protein (U/mL) to account for potential variations in sample composition. The observed enhancements in enzyme activities (e.g., duodenal trypsin: 349.79 vs. 220.10 U/mL in controls, *p* < 0.01) translate to practical benefits for commercial rabbit production: (1) improved feed conversion ratio (4.89 vs. 5.18 in controls) could reduce feed costs, and (2) the 56.2% lower diarrhoea incidence would significantly decrease mortality rates and medication use, as diarrheal diseases account for >70% of preweaning losses in commercial farms [15]. These economic advantages, combined with CA’s GRAS (generally recognised as safe) status, support its adoption as a sustainable alternative to antibiotic growth promoters in rabbit farming.

### 4.4. Effects of Dietary CA Supplementation on Intestinal Morphology of Meat Rabbits

As the main digestive organ in rabbits, the intestines play a critical role in nutrient digestion, absorption, and utilisation. The morphological integrity of the intestinal mucosa is also a prerequisite for digestive function [45,46]. Villi and crypts are important components of the small intestine, and their height and depth, respectively, reflect the absorptive capacity of the intestine [47]. An increased villus height improves surface area for absorption, and a lower crypt depth reflects a greater ability of intestinal stem cells to rapidly self-renew and repair [48]. In this study, we observed that dietary CA supplementation improved villus height in the duodenum, consistently with the findings of a previous study that demonstrated that dietary supplementation with a mixture of glycerol monolaurate and CA in laying hens significantly increases intestinal villus height, villus height/crypt depth, and duodenal villus area [49]. It has also been reported that supplementation with combined plant essential oils, namely lauric acid monoglyceride and CA at 350 and 500 mg/kg, respectively, enhances intestinal morphology in broiler chickens [50]. Our findings align with but exceed previous rabbit studies using phytogenic additives: the 11.7% increase in duodenal villus height (1111.80 vs. 994.99 μm) surpasses the 5–8% improvements reported for thymol [19] and eugenol [18] at comparable doses. This structural enhancement correlates with functional benefits, as each 100 μm increase in villus height improves amino acid absorption by ~18% in rabbits [45], suggesting our observed morphological changes could enhance nutrient uptake by approximately 21%. These synergistic effects explain the superior growth performance in CA-supplemented groups compared to other phytogenic compounds tested in rabbit nutrition studies.

### 4.5. Effects of Dietary CA Supplementation on Caecal Microflora Diversity of Meat Rabbits

A healthy intestinal flora can promote nutritional metabolism, growth, and development of the body while improving immunity and establishing a symbiotic relationship between intestinal flora and host [51]. Diarrhoea, primarily caused by intestinal flora imbalance, accounts for 75% of rabbit mortality [52]. The present study demonstrated that dietary CA supplementation significantly modulated caecal microbiota composition in the test animals, suggesting that it has potential, as a phytogenic feed additive, to enhance gut health. These findings align with those of previous studies, which indicated that phytogenic compounds such as CA exhibit selective antimicrobial activity against pathogenic bacteria while promoting beneficial microbial populations [46,53]. The enhanced microbial diversity in CA-supplemented groups further support its role in stabilising gut microbiota homeostasis and potentially mitigating dysbiosis-related metabolic disorders [54]. This conclusion further aligns with reports that CA, because of its antimicrobial properties, can reduce the population of opportunistic pathogens (such as *Escherichia coli*) while fostering the growth of commensal bacteria [55,56]. Such shifts may explain the improved nutrient digestibility observed in rabbits fed the CA-supplemented diet, as a balanced microbiota enhances fibre fermentation and energy utilisation [57]. However, the dose-dependent effect of CA requires further investigation given that excessive supplementation may disrupt intestinal microbial balance [58], consistently with the high diarrhoea and mortality rates observed in Group E rabbits, which were administered the high CA dose (200 mg/kg). Additionally, further studies are required to evaluate the long-term effects of CA on microbial resilience and host metabolism to optimise its application in rabbit nutrition.

CA supplementation significantly altered caecal microbiota by increasing beneficial fibre-fermenting bacteria (e.g., Oscillospiraceae +38%) with *p*-values FDR-corrected (q < 0.10). These changes enhanced villus growth (+11.7%), directly correlating with improved feed efficiency (FCR −5.6%) and reduced diarrhoea incidence (−56.2%), demonstrating CA’s dual role in promoting gut health and growth performance.

No significant differences in feed intake (ADFI: 179.72–182.06 g/d, *p* = 0.971) or palatability were observed across groups (Table 2), suggesting CA’s flavour-masking formulation (dextrin carrier) effectively neutralised potential odour/taste aversion at ≤200 mg/kg doses.

While this study demonstrated CA’s benefits on gut health and growth performance, its limitations include the inference of microbial functional potential via PICRUSt2 (phylogenetic investigation of communities by reconstruction of unobserved states) rather than direct metatranscriptomic analysis, along with unexamined proteomic changes in intestinal mucosa. Future studies should validate metabolic pathways (e.g., butyrate synthesis) through metabolomics, characterise host–microbe interactions via intestinal proteomics, and assess CA’s efficacy under diverse commercial farming conditions to bridge these mechanistic and translational gaps.

## 5. Conclusions

The results of this study showed that dietary supplementation with 100 or 150 mg/kg CA significantly improved the growth performance, antioxidant capacity, and intestinal health of meat rabbits, with the optimal CA dose (150 mg/kg) significantly enhancing nutrient utilisation by improving digestive enzyme (protease, amylase, and lipase) activity and duodenal villus length, while lowering diarrhoea incidence and mortality. Further, the levels of serum antioxidant markers (SOD, GSH-PX, CAT, and TPX) were markedly elevated following CA supplementation, indicating enhanced oxidative stress resistance. CA also modulated caecal microbiota by enriching fibre-fermenting bacteria (*Oscillospiraceae*) and suppressing potential pathogens (*Ruminococcaceae*). While this study demonstrated that dietary CA supplementation (150 mg/kg) enhances intestinal health and growth performance in meat rabbits through synergistic improvements in digestion, antioxidant capacity, and microbial ecology, two limitations should be noted: (1) the 47-day trial period may not fully reflect long-term effects, and (2) meat quality parameters (e.g., pH, shelf-life, sensory traits) were unassessed. Future research should evaluate CA’s cost-effectiveness in commercial production systems and consumer acceptance of CA-fed rabbit meat to facilitate industry adoption. Nevertheless, these results position CA as a promising phytogenic alternative to antibiotic growth promoters in rabbit farming.

## Figures and Tables

**Table 1:** Composition and nutrient profile of basal diets.

Items	Content (%)
Ingredients	
Corn	30
Soybean meal	15
Wheat bran	25
Peanut shell powder	5.0
Alfalfa meal	20.0
Premix ^1^	5
Total	100.00
Nutrient levels	
CP	17
DE/(MJ·kg^−1^)	10.6
CF	13
NDF	32
ADF	19
ADL	5
Ca	1
P	0.5

^1^ The premix provided the following per kg of the concentrate: Lys 1, 500 mg; Met, 1500 mg; CaHPO4, 15,000 mg; NaCl, 5000 mg; VA, 10,000 IU; VD3, 2000 IU; VE, 50 mg; VK3, 2.5 mg; thiamine, 5 mg; VB12, 1 mg; riboflavin, 10 mg; pantothenic acid, 50 mg; nicotinic acid, 20 mg; folic acid, 2.5 mg; choline chloride, 400 mg; Fe (as ferrous sulphate), 100 mg; Zn (as zinc sulphate), 50 mg; Cu (as copper sulphate), 40 mg; Mn (as manganese sulphate), 30 mg; I (as potassium iodide), 0.5 mg; Se (as sodium selenite), 0.05 mg.

**Table 2:** Effects of CA on growth performance of rabbits ^1^.

Items	CA, mg/kg					SEM	*p*-Value
	Group A 0	Group B 50	Group C 100	Group D 150	Group E 200		
IBW (g)	1103.36	1096.71	1097.33	1098.05	1081.64	8.017	0.956
FBW (g)	2525.26 ^BC^	2501.41 ^B^	2575.99 ^AC^	2624.42 ^A^	2592.50 ^A^	13.040	0.001
ADFI (g/d)	179.72	174.59	179.06	182.06	178.14	3.004	0.971
ADG (g/d)	34.68 ^B^	34.26 ^BC^	36.06 ^AC^	37.23 ^A^	36.85 ^A^	0.371	0.010
F/D	5.18	5.10	4.97	4.89	4.85	0.091	0.803
DR (%)	17.78 ^B^	14.44 ^B^	16.67 ^B^	7.78 ^C^	27.78 ^A^	1.766	<0.001
MR (%)	22.22 ^A^	16.67 ^BC^	13.33 ^C^	8.89 ^D^	20.00 ^AB^	1.366	<0.001

^1^ IBW, initial body weight; FBW, final body weight; ADG, average daily gain; ADFI, average daily feed intake; F/D, feed conversion ratio; DR, diarrhoea rate; MR, mortality rate; SEM, standard error of the mean. Within a row, values with different superscript letters differ significantly (*p* < 0.05, one-way ANOVA with Tukey’s test).

**Table 3:** Effects of CA on serum antioxidant capacity and lipid indices of rabbits ^1^.

Items	Group A	Group C	Group D	SEM	*p*-Value
SOD (U/mL)	56.09 ^C^	64.96 ^B^	74.20 ^A^	1.373	<0.001
GSH-PX (U/mL)	216.65 ^C^	239.28 ^B^	258.70 ^A^	3.017	<0.001
CAT (U/mL)	56.90 ^C^	61.99 ^B^	67.97 ^A^	0.837	<0.001
TPX (U/mL)	74.13 ^C^	86.14 ^B^	95.33 ^A^	1.564	<0.001

^1^ SOD, superoxide dismutase; GSH-PX, glutathione peroxidase; CAT, catalase; TPX, thioredoxin peroxidase; SEM, standard error of the mean. Data are presented as mean. Within a row, values with different superscript letters differ significantly (*p* < 0.05, one-way ANOVA with Tukey’s test).

**Table 4:** Effects of CA on intestinal digestive enzyme activities in meat rabbits ^1^.

Items	Tissue	CA, mg/kg			SEM	*p*-Value
		Group A 0	Group C 100	Group D 150		
Protease (U/mL)	Caecum	201.48 ^B^	237.46 ^A^	252.93 ^A^	6.25	<0.001
Amylase (U/L)		65.39 ^C^	77.79 ^B^	94.66 ^A^	2.99	<0.001
Lipase (U/L)		147.31 ^C^	172.12 ^B^	211.18 ^A^	6.39	<0.001
Protease (U/mL)	Duodenum	220.10 ^C^	286.26 ^B^	349.79 ^A^	12.38	<0.001
Amylase (U/L)		84.18 ^B^	96.86 ^B^	113.57 ^A^	3.08	<0.001
Lipase (U/L)		98.74 ^C^	122.11 ^B^	150.51 ^A^	5.28	<0.001

^1^ SEM, standard error of the mean. Data are presented as mean. Within a row, values with different superscript letters differ significantly (*p* < 0.05, one-way ANOVA with Tukey’s test).

**Table 5:** Effects of CA on the intestinal morphology of rabbits ^1^.

Items	Tissue	CA, mg/kg			SEM	*p*-Value
		Group A 0	Group C 100	Group D 150		
Villus length (μm)	Duodenum	994.99 ^B^	1003.48 ^B^	1111.80 ^A^	19.909	0.022
Crypt depth (μm)		114.32	119.97	119.93	3.340	0.746
Villus length/crypt depth (V/C)		8.84	8.68	9.58	0.273	0.350
Muscular thickness (μm)	Colon	175.82	175.24	166.30	5.326	0.746
Intestinal gland depth (μm)		383.78	381.14	340.69	8.819	0.076
Muscular thickness (μm)		411.34	432.44	409.56	11.002	0.643

^1^ SEM, standard error of the mean. Data are presented as mean. Within a row, values with different superscript letters differ significantly (*p* < 0.05, one-way ANOVA with Tukey’s test).

**Table 6:** Effects of CA on the alpha diversity of caecal microflora of rabbits ^1^.

Items	Group A	Group C	Group D	SEM	*p*-Value
OTU	916.67	928.33	932.83	5.048	0.428
Ace	1013.20	1005.10	1053.50	5.504	0.030
Chao	1018.50 ^B^	1015.50	1063.20 ^A^	5.938	0.019
Coverage (%)	99.28 ^B^	99.32 ^B^	99.51 ^A^	0.018	0.005
Shannon	5.21 ^A^	5.01 ^B^	5.24 ^A^	0.018	0.110
Simpson	0.02 ^B^	0.03 ^A^	0.02 ^B^	0.001	0.021
Sobs	871.17 ^B^	861.50 ^B^	941.83 ^A^	4.487	<0.001

^1^ SEM, standard error of the mean. Data are presented as mean. Within a row, values with different superscript letters differ significantly (*p* < 0.05, one-way ANOVA with Tukey’s test).

## Data Availability

The raw datasets used and analysed in this study are available from the corresponding author on reasonable request.

## Abbreviations

CA	Cinnamaldehyde
IBW	Initial body weight
FBW	Final body weight
AGPs	Antibiotic growth promoters
ADG	Average daily gain
ADFI	Average daily feed intake
FCR	Feed conversion ratio
SOD	Superoxide dismutase
GSH-PX	Glutathione peroxidase
CAT	Catalase
TPX	Thioredoxin-dependent peroxidase
DR	Diarrhoea rate
MR	Mortality rate
